# P03-02 Benefits of play that include physical activity in preschoolers on their adaptive functioning

**DOI:** 10.1093/eurpub/ckac095.038

**Published:** 2022-08-29

**Authors:** Rodrigo Gallardo, Laura Léniz, Karen Gallardo, Orlando Gallardo

**Affiliations:** 1 Department of Sports Science and Physical Conditioning, Catholic University of the Most Holy Conception, Concepción, Chile; 2 Independent researcher, Concepción, Chile; 3 Department of Physical Education, Faculty of Education, University of Concepción, Concepción, Chile

**Keywords:** children development, play, adaptive-functioning, preschoolers

## Abstract

**Background:**

Physical activity promotes the better physical and emotional well-being of children. A high percentage of Chilean children do not reach the minimum daily time expected of physical activity. Parents and educators of preschoolers are fundamental to the development of activities that involve movement. This study aimed to examine the relationship between the weekly time that Chilean preschoolers include physical activity in their plays and children's development. Also, compare child development between preschoolers who engaged in physical activity play with or without caregivers' presence.

**Methods:**

The sample consisted of 54 preschoolers aged 34.7-65.9 months (mean 52.0±10.2). The parents completed: a) Ages and Stages Questionnaire Third Edition to measure communication, motor, solving-problem, and personal-social development, and Ages and Stages Questionnaire Socioemotional to measure the socio-emotional development of children and their areas (self-regulation, compliance, autonomy, adaptive functioning (physiological needs), affection, social communication, and interaction; b) an ad-hoc questionnaire to register the time that children dedicate weekly to physical activity on their play, and if their children engaged in play that included physical activity with their caregivers or not.

**Results:**

Pearson coefficient showed that preschoolers' time spent weekly on plays that included physical activity was moderate and significant (p > 0.05) when related to social communication's, adaptive-functioning's, personal-social's, and communication's development. T Student for independent samples revealed that Self-regulation (t=-2.09; p > 0.05) and adaptive-functioning's development (t=-2.99; p > 0.01) were better in preschoolers engaged in play that included physical activity with their caregivers than those who did not play with them. A multivariate regression analysis indicated that preschoolers' adaptive-functioning could be predicted by a combination of higher time dedicated weekly to physical activity on their play and children playing with their caregivers.

**Conclusions:**

These findings suggest that physical activity in preschoolers could benefit their physiological needs. Thus, it is necessary to promote strategies that include the family and increase physical activity time on children's plays.

